# A Novel Inchworm-Inspired Soft Robotic Colonoscope Based on a Rubber Bellows

**DOI:** 10.3390/mi13040635

**Published:** 2022-04-17

**Authors:** Jinyan Chen, Jianlin Yang, Feng Qian, Qing Lu, Yu Guo, Zhijun Sun, Chao Chen

**Affiliations:** 1 State Key Laboratory of Mechanics and Control of Mechanical Structures, Nanjing University of Aeronautics and Astronautics, Nanjing 210016, China; jychen@nuaa.edu.cn (J.C.); yangjianlin@nuaa.edu.cn (J.Y.); nuaa_qf@nuaa.edu.cn (F.Q.); lq331572639@163.com (Q.L.); 2 College of Mechanical and Electrical Engineering, Jinling Institute of Technology, Nanjing 211169, China; guoyu0834@163.com

**Keywords:** colonoscopy, soft robot, inchworm inspired

## Abstract

Colorectal cancer is a serious threat to human health. Colonoscopy is the most effective procedure for the inspection of colorectal cancer. However, traditional colonoscopy may cause pain, which can lead to the patient’s fear of colonoscopy. The use of active-motion colonoscopy robots is expected to replace traditional colonoscopy procedures for colorectal cancer screening, without causing pain to patients. This paper proposes an inchworm-like soft colonoscopy robot based on a rubber spring. The motion mechanism of the robot consists of two anchoring units and an elongation unit. The elongation unit of the robot is driven by 3 cables during contraction and by its inherent elasticity during extension. The balloon is selected as the anchoring mechanism of the robot. It has soft contact with the colon and will not damage the colon wall, which means no discomfort is caused. The elastic force test of the rubber spring shows that the elongation unit of the robot has sufficient restorative force to drive the robot to move forward and backward. The influence of the balloon’s expansion size on the dexterity of the robot head is analyzed, and the functions of the balloons are expounded. The balloon can not only assist the robot in its locomotion but also assist the robot to perform a better inspection. The robot can move successfully in a horizontal, straight, and inclined isolated pig colon, showing great clinical application potential.

## 1. Introduction

The incidence rate of colorectal cancer ranks third among all cancers, representing a serious threat to human health [1]. At present, colonoscopy is the main means of colorectal cancer screening and the gold standard [2]. However, colonoscopists require a long period of training to skillfully complete the colonoscopy processes using traditional colonoscopes. In a colonoscopy procedure, a traditional colonoscope is pushed into the intestine, which will stretch the intestine and make the patients feel pain, and may lead to complications such as bleeding and perforation. Thus, patients are usually reluctant to receive a colonoscopy. Therefore, a safer and compliant device is needed to replace the push-type traditional colonoscopy.

Active-locomotion or self-propelling robotic colonoscopes are expected to replace colonoscopists in the examination of human intestines, a field that has attracted many researchers in the last two decades. Such robotic colonoscopes can actively move forward and turn in the colon, and can even complete other tasks that can be completed via traditional colonoscopy, such as biopsy. At present, robotic colonoscopes are mainly divided into the following categories: magnetic-driven [3,4], wheeled [5,6,7,8], legged [9,10,11,12,13], peristaltic [14,15], inchworm-like [10,16,17,18,19,20,21,22,23,24,25], and other kinds of robots [26,27,28]. These robots have made great contributions to the development of colonoscopy robots. However, due to the complex environment in the colon, almost no colonoscopy robot has reached the stage of clinical application, except the Endotics System [16].

Among the colonoscopy robots mentioned above, the motion of inchworm-like colonoscopy robots is relatively stable, and the soft inchworm-like colonoscopy robots can conform to the shape of and make flexible contact with the colon, which is safer. Generally, an inchworm-like colonoscopy robot includes two anchoring units, an elongation unit, and a steering unit. Inchworm-like robots such as the Endotics System use a vacuum suction device and a gripper as the anchoring unit, a pneumatic actuator as the elongation unit, and a memory alloy actuator or a pneumatic actuator as the steering unit [16,17]. They allow stable anchoring, large strokes, and flexible steering, and have achieved good experimental and clinical results. Another kind of inchworm-like robot utilizes a balloon, which is a simple structure, as the anchoring unit and a multi-degree-of-freedom pneumatic actuator with steering ability as the extension unit [18,19,20]. There are also other devices with great mobility and dexterity that use pneumatic actuators for endoscopes or inchworm-like robots [21,22,23,24,25,26]. However, the pneumatic actuator is driven by relatively high-pressure gas, which easily leaks. The movement efficiency of the actuator is relatively low, as the gas is transmitted through a long and narrow pipe. Another inchworm-like robot utilizes shap memory allor (SMA) to drive the robotic colonoscope, although the system is slow and has a reduced stroke [27]. The endoscopes equipped with inchworm-like assistance devices have relatively large diameters [28,29]. Chen et al. developed a self-propelling inchworm-like endoscopic device with novel structures, although the stroke of the propulsion segment and the expanding diameter of the grippers were both insufficient [30]. Bernth et al. developed a cable-driven robotic mesh worm actuated by onboard DC motors, although this robot, having a large diameter, was not suitable for the colon environment [31]. Overall, most of the existing inchworm-like colonoscopy robots have not had sufficient hollow space for the passage of all functional devices used in traditional colonoscopy, especially when the size of the robot is small enough. It is difficult to ensure that the robot has enough driving force if the hollow space of the robot is large.

In this paper, an inchworm-like robotic colonoscope is proposed, which uses the natural elastic force of a rubber spring and cables for elongation and contraction, as well as balloons for anchoring. The rubber bellows not only have enough rebounding force to drive the robot forward but also have a large hollow space for the passage of functional devices. The robot can perform all functions of a traditional colonoscopy instrument, such as biopsy, gas insufflation, waterjet procedures, and so on. Additionally, the robot has the advantages of having a simple structure and low cost. Our experimental results show that the robot can move forward successfully in an isolated pig colon, showing great potential for future therapeutic applications.

## 2. Design and Analysis of the Robot

### 2.1. Design Requirements

To design a colonoscopy robot, the first factor that should be considered is the size of the robot. The human colon mainly consists of six parts: the cecum, ascending colon, transverse colon, descending colon, sigmoid colon, and rectum. Their inner diameters are between 26 and 45 mm [32]. Therefore, the diameter of the robot should not exceed the minimum inner diameter of the colon. To pass through the anus more smoothly, the diameter of the robot should be as small as possible. The robot should also have the ability to change its diameter to adapt to colon sections with different inner diameters to obtain sufficient traction force. Another key consideration is the safety of the robot. The robot cannot have sharp and hard edges, as these may damage the fragile intestines and lead to accidents such as bleeding and perforation. The robot should also be dexterous enough to move forward and turn in complex colon environments. The head or distal end of the robot should be steerable to obtain the best view angle of inspection. The robot also needs to have a certain forward speed to ensure the inspection efficiency and complete the inspection process within the specified timeframe. In addition, to replace traditional colonoscopy, it should also be able to perform all functions of a traditional colonoscopy device, such as biopsy, gas insufflation, waterjet processes, and so on. In short, the robot should meet the requirements across five aspects: size, safety, dexterity, speed, and functionality.

### 2.2. The Overall Design of the Robot

**Figure 1:**
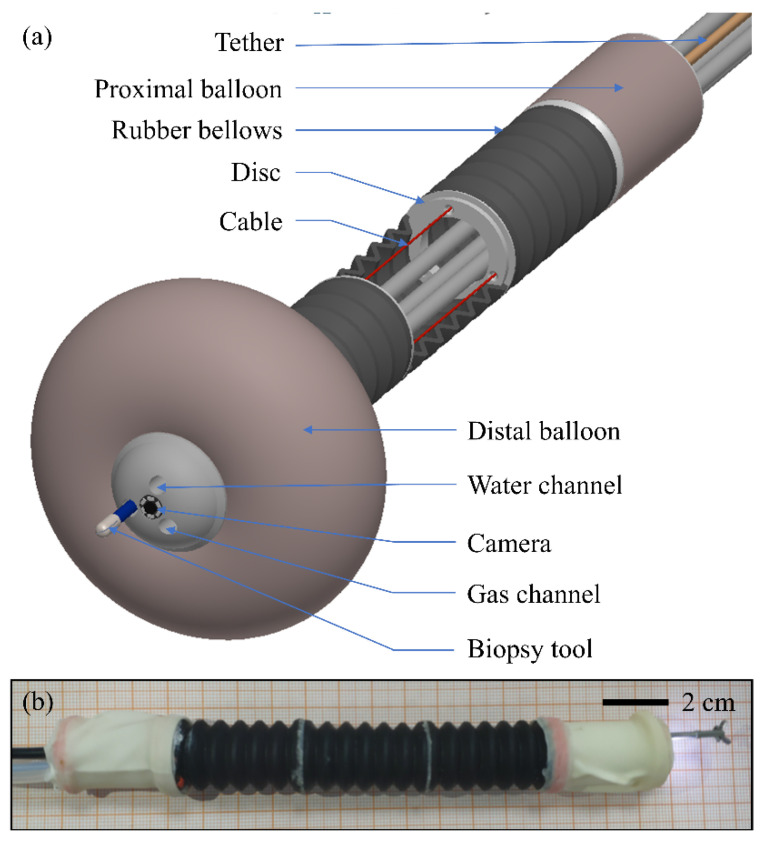
(**a**) CAD drawing of the robot. (**b**) Prototype of the robot.

As is shown in Figure 1a, the locomotion mechanism of the robot mainly includes three parts: two anchoring units and an elongation unit. The elongation unit can extend and contract along its axis and can bend around two axes orthogonal to the axis of the robot. The elongation unit is composed of several rubber bellows modules that are connected by discs. The small disc is circular, with three small holes symmetrically distributed along the circumference, while a cable passes through the small hole. The wires, channels of functional devices and air supply tube to the distal balloon can pass through the disc center. The function of the disc is that if the ratio of length over the diameter of the bellows is too large, it will lead to axial instability of the rubber bellows after compression. Therefore, discs are required, so that the length and diameter of each bellow module are relatively small and instability will not occur. The extension of the elongation unit depends on the inherent elasticity of the rubber bellows, and the contraction and bending depend on the three cables. If the three cables are pulled at the same distance, the elongation unit will only contract along its axis. If the three cables are pulled at different distances, the bellows will bend while contracting. The bellows can be regarded as a length-variable continuum mechanism. The balloon is widely used in the clinic [33], especially during lumen inspection. The balloon has good flexibility to adapt to the complex colon environment, and is cheap and easy to obtain. Hence, the balloon is selected as the anchoring unit to provide anchorage for the robot. A camera, a biopsy tool, a waterjet channel and a gas channel are installed in the distal anchoring unit. In other words, the robot also has the functional devices of traditional colonoscopy. The miniature camera (DN-3.9, Dannan Technology, Shenzhen, China) is 4 mm in diameter with illuminating LEDs on its end. In addition, there is a tether behind the robot, which is composed of air tubes, channels, cables and PEEK tubes. Figure 1b shows the prototype of the robot and its specifications are shown in Table 1. The anchoring unit bodies at the proximal and distal ends of the robot and the discs are 3D-printed. The balloons and air tubes are composed of latex and silica gel and are bonded to the anchor units and ports, respectively. The bellows are made of rubber and manufactured by molding. The robot is low cost and can be disposable.

### 2.3. Working Principle of the Robot

**Figure 2:**
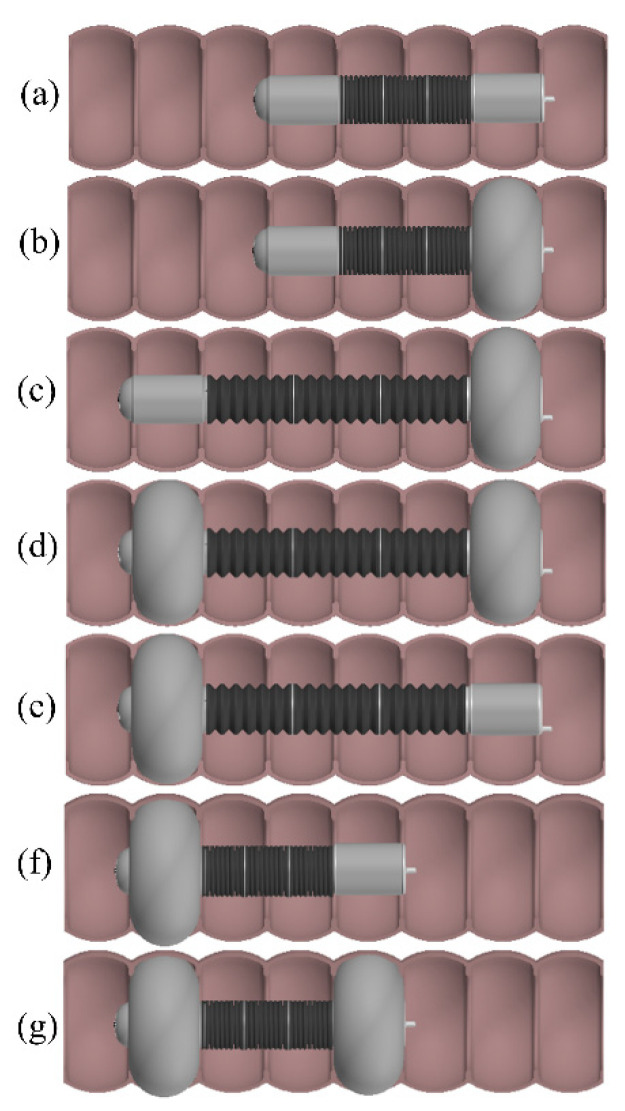
(**a**) Initial state. (**b**–**g**) Locomotion cycle sequences of the robot.

Figure 2 shows the locomotion sequences of the robotic colonoscopy. In the initial state (Figure 2a), the robot is contracted, and the proximal and distal balloons are not pressurized. In this state, the robot has relatively high rigidity and easily passes through the anus and enters into the colon. Next, the robot begins to move, as shown in Figure 2b–g:

(b) The proximal balloon is pressurized first to provide anchoring for the robot;

(c) The displacements of driving cables are adjusted, and the rubber spring in the middle of the robot will rebound, relying on its elastic force to drive the robot head forward. Meanwhile, the robot head can turn by adjusting the speeds of the three driving cables;

(d) The distal balloon is pressurized to provide anchorage for subsequent robot movement;

(e) The proximal balloon is depressurized and the anchorage between the balloon and the intestine is canceled. This process can be carried out simultaneously with the third step to improve the motion efficiency of the robot;

(f) The driving cables are pulled to compress the rubber spring to pull the proximal anchoring unit of the robot and the tether forward;

(g) The proximal balloon is pressurized and the pressure of the distal balloon is released, returning to the first step.

In this cycle, the robot can move forward like an inchworm. Acting in reverse order, the robot can move backward. For the colonoscopy robot with a tether, when it returns, the shape of the tether may be very complex, and the robot cannot push the tether out of the body by thrusting. Therefore, in general, the tether needs to be pulled by an external force to pull the robot out of the body.

### 2.4. Dexterity Analysis of Robot Head

The head dexterity of the robotic colonoscope has an important influence on the colonoscopy process. Firstly, it can assist the camera to obtain a wider field of vision, and even allow the robot to see the situation behind the robot. Secondly, in the process of biopsy or other surgical operations, the posture adjustment of the instrument also requires the movement and deflection of the robot head to assist. In addition, the deflection of the robot head can also assist the colonoscopy in moving forward and turning. Therefore, it was necessary to analyze the dexterity of the robot head. The inchworm-like colonoscopy robot has the same flexible continuum structure as traditional colonoscopy instruments. However, it has two balloons for anchoring, and its continuum structure can be compressed. This section focuses on the dexterity analysis of the inchworm-like colonoscopy robot in the colon and the influence of balloons on the dexterity of the robot head.

The robot must be able move in soft, slippery, and three-dimensional tortuous colon environments, which brought great difficulties to the analysis. To simplify the analysis, here the dexterity of the robot head in a straight colon is analyzed using a two-dimensional plane. At the same time, the following assumptions are made:

(1) Ignoring the influence of haustral folds in the colon, the colon is considered to have a smooth cylindrical inner wall;
(2) Like other continuum structural analyses, the elongation of the robot satisfies the constant curvature assumption;
(3) The influence of the tether behind the robot on the dexterity of the robot is not considered;
(4) The deformation of balloons and colon after stress is not considered; that is, the colon is regarded as rigid, and the balloon is regarded as a rigid body.

**Figure 3:**
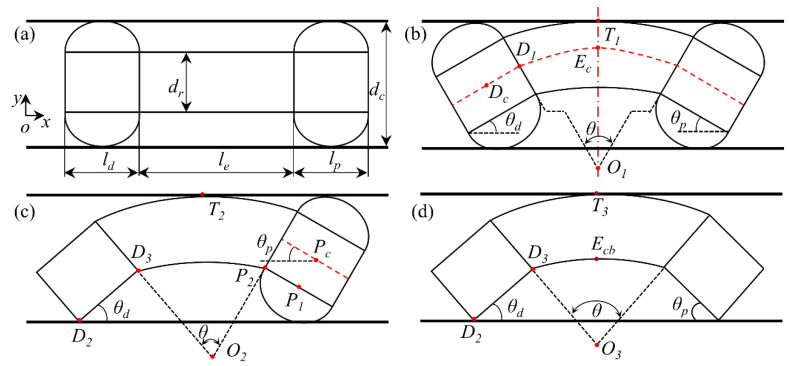
Schematic diagram of robot head flexibility analysis. (**a**) Both balloons are anchored and the robot is straight. (**b**) Both balloons are anchored and the robot is in the bending limit state. (**c**) Single balloon is anchored and the robot is at the bending limit state. (**d**) Both balloons are not anchored and the robot is in the bending limit state.

As shown in Figure 3a, both balloons are anchored. At this time, the distal and proximal anchoring units of the robot are in the center of the colon. In Figure 3a, $l_{d}$, $l_{e}$, $l_{p}$, $d_{c}$, and $d_{r}$ are the same length as the distal anchoring unit, elongation unit, proximal anchoring unit, colon diameter, and robot diameter, respectively. When the cables are driven to adjust the curvature of the elongation unit, the anchoring unit will deflect. In this process, there may be friction between the balloon and the colon. However, the pressure in the balloon can be adjusted so that there is only a small contact force between the balloon and the intestinal wall. Since the colon is very slippery, the friction between the balloon and the colon is very small and can be ignored. Thus, the friction will not affect the deflection of the anchoring device. After a certain angle of deflection, the geometric centers of the two anchoring devices are still on the centerline of the colon. The elongation unit continues to bend until it touches the intestinal wall. As shown in Figure 3b, the two anchoring units deflect $\theta_{d}$ and $\theta_{p}$, respectively. Thus, the length of the distal and proximal anchoring units is the same due to symmetry:

(1) $$\theta_{d}=\theta_{p}=\frac{1}{2}\theta$$

where θ is the included angle of the central axis of the two anchoring devices and is also the included angle of the two end faces of the extension device.

To make $\theta_{d}$ as large as possible, the curvature of the elongation unit should be as large as possible. In other words, from Figure 3b, the lower arc of the elongation unit should be as short as possible. The shortest length of the arc is considered the shortest length of the elongation unit, although there may be an error with the actual situation. According to the geometric relationship, the *y*-axis components of the three vectors $D_{c}D_{1}$, $D_{1}E_{c}$, and $E_{c}T_{1}$ satisfy:

(2) $$\frac{l_{ea}}{\theta }-\frac{l_{ea}}{\theta }\cos\frac{\theta }{2}+\frac{l_{d}}{2}\sin\theta_{d}+\frac{d_{r}}{2}=\frac{d_{c}}{2}$$

where $l_{ea}$ is the arc length of the center axis of the elongation unit, which satisfies:

(3) $$\frac{l_{ea}}{\theta }-\frac{l_{e\min}}{\theta }=\frac{d_{r}}{2}$$

where $l_{emin}$ is the shortest length of the elongation unit.

As is shown in Figure 3c, the gas in the distal balloon is gradually discharged, and the elongation unit continues to bend until the distal anchoring unit body touches the intestinal wall. In this configuration, the anchoring units at the distal end and proximal end have different deflection angles. The *y*-axis components of the three vectors $D_{2}D_{3}$, $D_{3}O_{2}$**,** and $O_{2}T_{2}$ satisfy:

(4) $$l_{d}\sin\theta_{d}-\frac{l_{e\min}}{\theta }\cos\theta_{d}+\frac{l_{e\min}}{\theta }+d_{r}=d_{c}$$

Here, *y*-axis components of the four vectors $P_{c}P_{1}$, $P_{1}P_{2}$, $P_{2}O_{2},$ and $O_{2}T_{2}$ satisfy:

(5) $$-\frac{d_{r}}{2}\cos\theta_{p}+\frac{l_{p}}{2}\sin\theta_{p}-\frac{l_{e\min}}{\theta }\cos\theta_{p}+\frac{l_{e\min}}{\theta }+d_{r}=d_{c}$$

According to the geometric relationship, it can be seen easily that $\theta_{d}$ and $\theta_{p}$ satisfy:

(6) $$\theta_{d}+\theta_{p}=\theta$$

Then, as shown in Figure 3d, the proximal anchoring balloon continues to be discharged and the elongation unit continues to bend until the proximal anchoring unit body touches the colon wall. The *y*-axis components of the three vectors $D_{2}D_{3}$, $D_{3}E_{cb},$ and $E_{cb}T_{3}$ satisfy:

(7) $$\frac{l_{e\min}}{\theta }-\frac{l_{e\min}}{\theta }\cos\frac{\theta }{2}+l_{d}\sin\theta_{d}+d_{r}=d_{c}$$

Here, $\theta_{d}$ and $\theta_{p}$ satisfy:

(8) $$\theta_{d}=\theta_{p}=\frac{1}{2}\theta$$

In these three cases, as the size of each part of the robot has been determined and the colon diameter is variable, the maximum rotation angle of the robot head $\theta_{d}$ is only related to the colon diameter. Therefore, the relationship between the maximum deflection angle of the robot head $\theta_{d}$ and the colon diameter $d_{r}$ in three cases can be obtained via simultaneous Equations (1)–(8), respectively. In another case, the proximal end of the robot is not anchored and the distal end is anchored, which is contrary to the case in Figure 3c. In this case, the maximum deflection angle of the robot head is the deflection angle of the proximal anchoring device shown in Figure 3c. This angle can also be obtained via simultaneous Equations (4)–(6). The dexterity angle of the robot head $D_{rh}$ is defined as twice the maximum deflection angle of the robot head; that is:

(9) $$D_{rh}=2\theta_{d}$$

This definition is similar to the literature [34]. The difference is that in our definition, the distal end of the robot is not fixed, but deviates from the radial and axial direction of the colon. The dexterity of the robot head mainly affects the view angle of the colonoscopy robot and the biopsy operation. The focal distance of the camera is variable, and the biopsy forceps can move in the channel. Moreover, the robot can move forward and backward in the colon. Therefore, the deviation of the distal end of the robot from the radial and axial directions of the colon will not affect the rationality of our definition.

**Figure 4:**
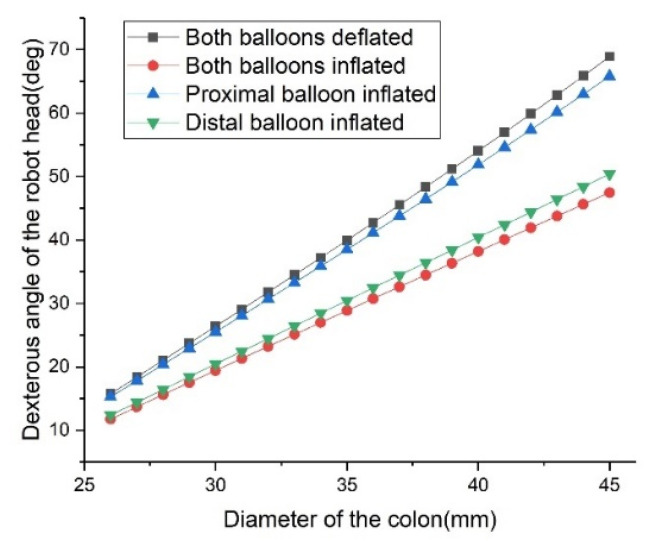
Relationship between robot head dexterity and colon diameter for four cases, regardless of whether the two balloons are inflated or not.

For these four cases, the relationships between robot dexterity angle and colon diameter are shown in Figure 4. It can be seen that when both balloons are inflated, the dexterity angle of the robot head is significantly smaller than that when both balloons are deflated. The influence of the distal balloon on the dexterity angle of the robot head is much greater than that of the proximal balloon. When the balloon is underinflated, i.e., when the balloon is not in full contact with the intestine, the dexterity angle of the robot head is between the case where the two balloons are fully inflated and the case where the two balloons are deflated. It is worth mentioning that this does not mean that the existence of balloons is a negative factor. On the contrary, the expansion diameter of the balloon can be adjusted so that the deflection angle of the robot head can change continuously within the maximum dexterity angle. For example, without balloons, it is difficult for the distal and proximal anchoring units of the robot to be both on the central axis of the colon and to obtain the best view angle. It can also be seen from the above description that the head of the robot can be stably and continuously deflected from the state in Figure 3a to the state in Figure 3d. Therefore, the balloon can not only assist the robot in its locomotion, but can also assist the robot to perform better inspections and even some surgical operations.

## 3. Test and Experiment

### 3.1. Test of the Movement Ability of the Robot

**Figure 5:**
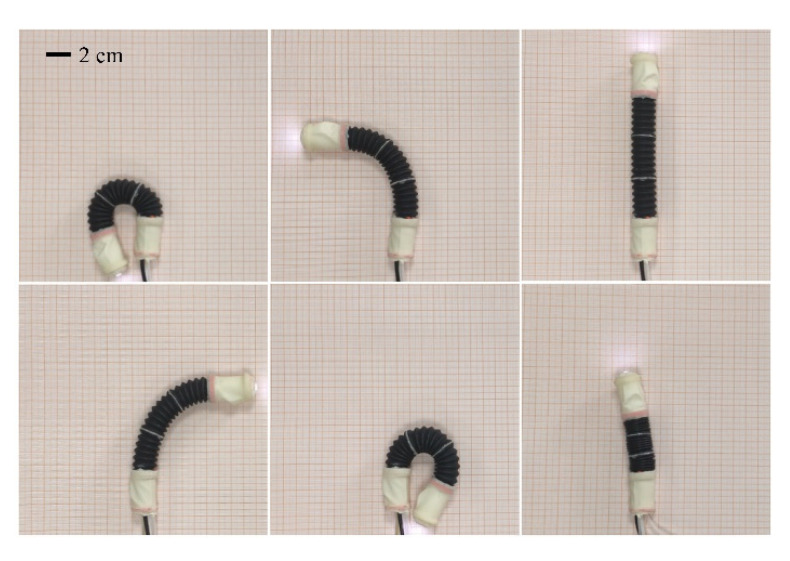
Bending, elongation, and contraction state diagram of the robot.

The bending ability is an important index to measure the motion ability of a soft inchworm robot. As shown in Figure 5, to test the bending, extension, and contraction ability of the robot, the robot is placed on a piece of paper with grid lines. The robot can bend more than 180° in both directions and the minimum radius of curvature is 20 mm, which means that the robot is competent for the task of moving in the three-dimensional tortuous complex colon. The length of the robot is 165 mm in the extended state and 115 mm in the contracted state. The length of the robot elongation unit is 105 mm, and the ratio of extension to contraction is close to 2:1.

### 3.2. Elastic Force Test of the Rubber Bellows

**Figure 6:**
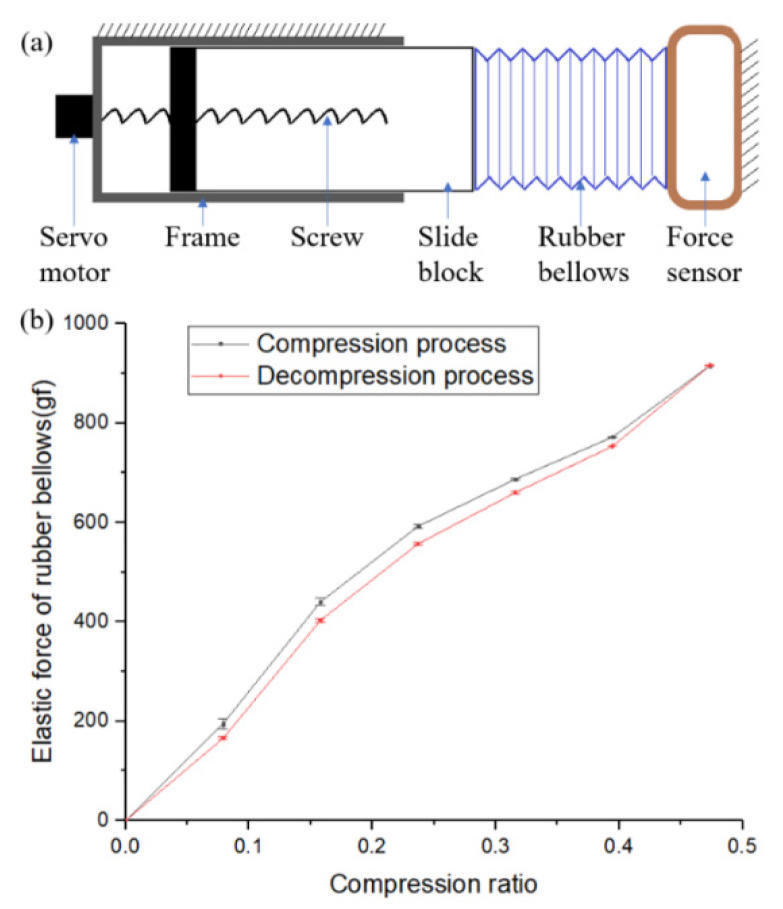
(**a**) Schematic diagram of rubber spring elasticity test. (**b**) Relationship between elastic force and compression ratio of the rubber spring.

The elastic force of the bellows is one of the driving forces of the robot, which has a very important impact on the motion performance of the robot. Hence, it is necessary to test the elastic force of the bellows. Figure 6a shows the schematic diagram of the elastic force test bench of the rubber bellow module of the elongation unit. The lead screw nut mechanism pushes the slide block and then pushes the bellows to compress. The other end of the bellows is against the force sensor and both ends of the bellows are connected with small discs. With this bench, the relationship between the elastic force and compression length of the bellows can be easily measured. It is a well-known fact that there will be stress relaxation in hyperelastic materials after deformation [35]. The rubber bellows also show obvious stress relaxation. Therefore, to obtain representative data, the bellows are repeatedly compressed and released quickly more than 100 times before the test. Due to stress relaxation, the bellows will be compressed or released to a certain length, and the elastic force of the bellows will change over time. Hence, the data from the force sensor are recorded 30 s after the bellows are compressed or released. Then, the bellows immediately continue to be compressed or decompressed to complete three cycles from compression to decompression. Finally, the relationship between the elastic force and the compression ratio is obtained, and the compression ratio of the bellows is the ratio of the compressed length to the original length of the bellows.

As shown in Figure 6b, it can be seen that the rubber bellows show stress relaxation; that is, the elastic force of the bellows in the compression process is a little larger than that in the release process. It can be seen that there is an approximately linear relationship between the elastic force and the compression ratio of the rubber bellows. The rubber bellows soften after more than 100 cycles of continuous compression and release; that is, the elastic force gradually decreases. Even so, when the compression ratio is 0.1, the elastic force of the bellows can still be over 200 gf. This means that when the 105-mm-long elongation unit of the robot is compressed by only 10 mm, it still has a rebounding force of nearly 2 N. The resistance to being overcome by the head of the robot in the process of moving forward in the colon almost only comes from friction and gravity. There is a large amount of mucus in the intestinal tract, which is very wet and slippery, so the friction force between the robot head and the intestinal tract is very small when it is not anchored. Therefore, the elasticity of the bellows is enough for the robot to move forward in the intestines. This can be verified in subsequent experiments. In addition, through the same test device, the bellows can withstand 1000 consecutive extreme compression and rebound cycles without obvious failure. Therefore, the bellows allow disposable use.

### 3.3. In Vitro Test

**Figure 7:**
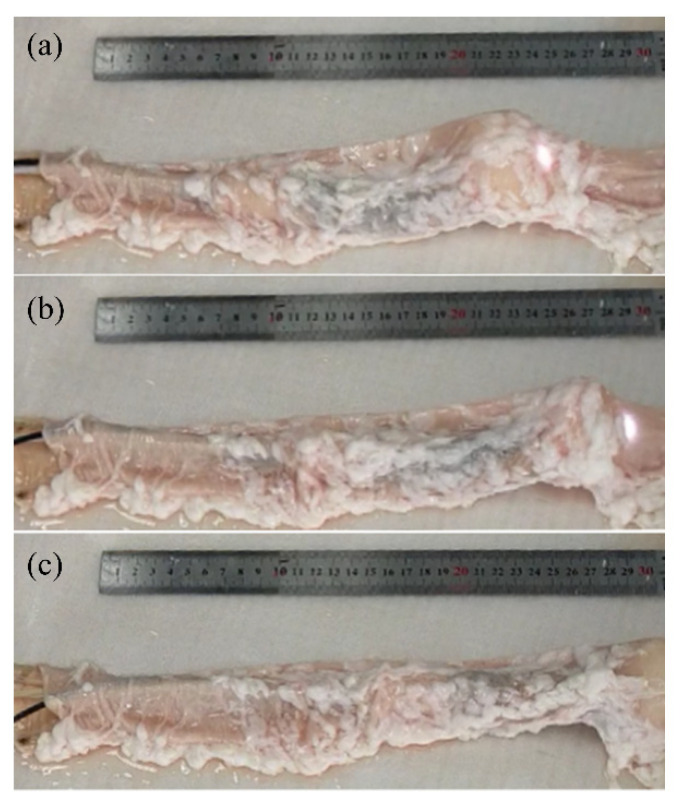
Locomotion of robot in the horizontal straight colon. The robot moves form (**a**) to (**b**) and then to (**c**).

In order to verify the movement feasibility of the robot in the actual intestine, a fresh pig large intestine (Suguo Supermarket, Nanjing) was prepared. An experiment of movement in an isolated pig intestine was carried out. As is shown in Figure 7, both ends of the colon segment used for the test were fixed. The colon was straightened and laid flat on the panel. Since the robot was not equipped with a motor, the robot was controlled manually. In the actual experiment, the robot was controlled by two people—one controlled the pulling and releasing of three cables, and the other one controlled the expansion and contraction of balloons at both ends. The expansion and contraction of the balloons were achieved via the inflation and extraction of the air cylinder. After the robot worked for several cycles, we assessed whether the anchoring device of the robot being anchored or not greatly affected the motion efficiency of the robot. If the balloon at the head or tail did not expand enough, it would slide when the robot contracted or extended. However, if the balloon expanded enough, the robot would barely slip. The colon locomotion test in Figure 7 represented the selected two cycles in which the anchoring balloon could effectively anchor. It was found that when the proximal anchoring balloon was anchored, the elongation unit of the robot could extend quickly. The elongation unit could extend almost to the original length, which proved that the resistance on the robot head was small and the rebounding force of the rubber bellows was sufficient. In these two cycles, the actual strokes of the robot were 43 mm and 46 mm, respectively, which were close to the experimental results and theoretical analysis results in the literature [36].

**Figure 8:**
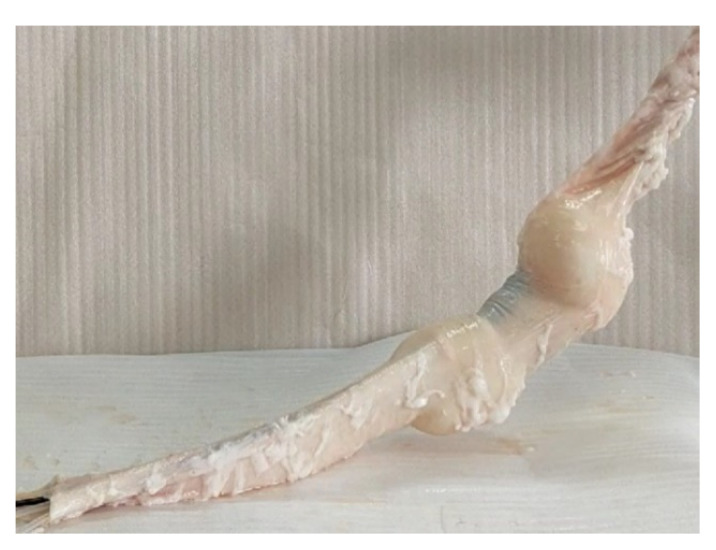
Movement of robot in the inclined pig colon.

Considering that the colon in the actual human body is a three-dimensional tortuous organ, the movement ability of the robot was also tested in the inclined colon. As shown in Figure 8, one end of the colon was fixed on the upper right panel and the other end was fixed on the horizontal panel. In the human colon, the sigmoid colon has a slope, so the isolated pig colon was used to simulate this part. To pass through this section, the robot not only had to overcome its gravity but also had to move forward and turn in this non-fixed intestinal section, which was very challenging. In fact, when the robot passed through this section of the intestine, the efficiency of the robot did decrease. However, the robot could still successfully pass through the intestinal segment, which showed the motion validity of the robot in this case. The motion efficiency of the robot was relatively high when the slope was small, but the motion efficiency of the robot decreased significantly with the increase in the slope. The main reasons were that firstly the gravity component to be overcome by the robot became larger because the slope increased. Secondly, due to the bending deformation of the tether and the increase in its length in the colon, the friction of the tether behind the robot (including wires, PEEK tubes, and air tubes) also increased, which was also an important reason for the decline of the motion efficiency of the robot.

## 4. Conclusions and Future Work

We have presented a soft inchworm-like colonoscopy robot using rubber bellows as a spring. As one of the driving power sources, the rubber spring can not only provide sufficient driving force, but can also provide a large hollow space for the installation of functional devices such as the air supply channel, water supply channel, biopsy forceps channel, and wires. Through the elastic force test, we find that the rubber bellows were shown to have sufficient rebounding force as the forward power of the robot. The effects of the balloons on the inchworm-like robot were also analyzed, and the results showed that whether the balloons are inflated at the proximal and distal ends has a different influence on the dexterity of the robot head. Combined with the bending of the elongation unit of the robot, the balloons can assist the robot head to deflect continuously within the maximum dexterity angle, which will ensure the stability of the robot camera and the robustness of surgical operations in the future. The proposed inchworm-like robot has a stroke of 50 mm and bends more than 180 degrees, which shows its potential to move in three-dimensional tortuous colon environments. Moreover, the motion capability of the robot in the isolated pig colon was experimentally assessed. The results show that the robot acn move forward successfully in the horizontally straightened pig colon and the inclined pig colon. Thus, the colonoscopy robot has very promising clinical application prospects.

Future work will focus on the construction of the robot, including the integration of necessary actuators and sensors, such as servomotors for driving the cables, air pressure sensors for detecting the pressure in the balloons, and a positioning sensor for obtaining the pose of the robot. Moreover, combined with real-time images captured by a camera, the robot is further expected to move automatically, meaning the performance of the robot can be improved.

## Figures and Tables

**Table 1:** Specifications of a fabricated robot.

Parameter	Dimension
Diameter of the robot	20 mm
Diameter of the hollow space of the robot	12 mm
Length of robot in the extended state	165 mm
Length of robot in the contracted state	115 mm
Length of the proximal anchoring unit	30 mm
Length of the distal anchoring unit	30 mm
Inner diameter of the rubber bellows	14 mm
Inner diameter of the disc	12 mm
Maximum expanded diameter of the balloon	75 mm
Diameter of the camera	4 mm
Length of the camera	28 mm
The weight of robot	30 g
